# *Staphylococcus lugdunensis* Endocarditis Presenting with Brain Abscesses and Temporal Vision Deficits

**DOI:** 10.1155/2024/4728700

**Published:** 2024-04-20

**Authors:** Matthew S. Linz, Diana Finkel, Eli S. Goshorn

**Affiliations:** ^1^Rutgers New Jersey Medical School, Newark, NJ 07103, USA; ^2^Division of Infectious Diseases, Department of Medicine, Rutgers New Jersey Medical School, Newark, NJ, USA

## Abstract

*Staphylococcus lugdunensis* is a coagulase-negative staphylococcal bacterium (CoNS) that colonizes the skin. While infectious endocarditis (IE) caused by *S. lugdunensis* is rare, it is noteworthy because it has been associated with an aggressive clinical course. In this report, we present a case of culture-negative IE complicated by brain abscesses, vision deficits, and progressive heart failure that ultimately required mitral valve replacement. The causative agent was eventually identified as *S. lugdunensis* through molecular testing of valvular tissue.

## 1. Introduction

Most CoNS species are considered to be less virulent than *Staphylococcus aureus*; however, in recent years, *Staphylococcus lugdunensis* has emerged as a human commensal CoNS bacterium with invasive pathogenic ability [1]. While *S. lugdunensis* is reported to cause less than 1% of native valve IE cases, IE due to *S. lugdunensis* is noteworthy because it is associated with an aggressive and morbid disease course. Mitral valve disease is most common, often progressing rapidly to destruction of valve tissue, onset of heart failure, and need for valve replacement. Moreover, the incidence of *S. lugdunensis* IE has likely been underestimated because of the use of latex agglutination tests that agglutinate both *S. aureus* and *S. lugdunensis*; implementation of matrix-assisted laser desorption ionization-time of flight mass spectrometry (MALDI-TOF MS) for species identification increased identification of *S. lugdunensis* in clinical isolates by a factor of 18 in one study [2]. We present the case of an adult male presenting with invasive *S. lugdunensis* endocarditis causing abscesses in the brain, spleen, and kidneys that progressed to heart failure requiring mitral valve replacement.

## 2. Case Presentation

**Figure 1:**
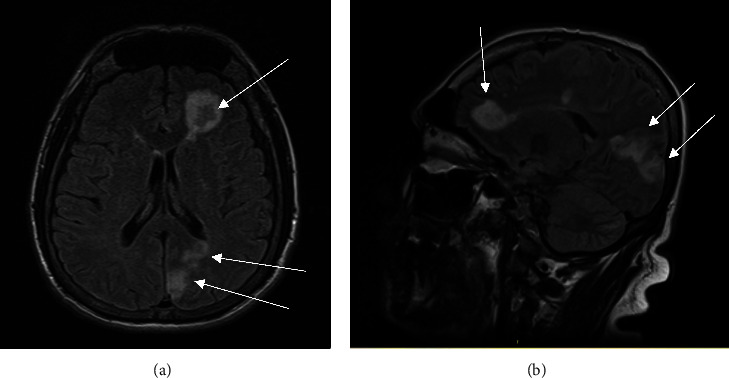
(a) Axial and (b) sagittal views of initial MRI imaging displayed left frontal, occipital, and parietal lobe abscesses with leptomeningeal enhancement.

**Figure 2:**
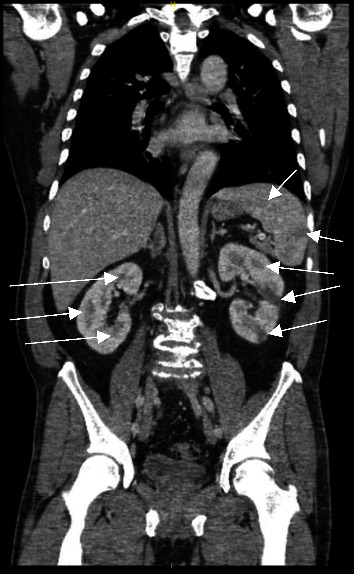
The patient's initial CT scan of the chest, abdomen, and pelvis was remarkable for infarcts in the spleen and bilaterally in the kidneys.

**Figure 3:**
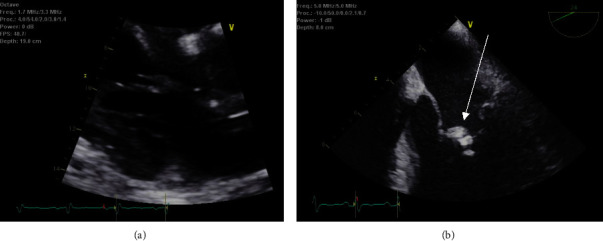
(a) Transthoracic echocardiography (TTE) and (b) follow-up transesophageal study (TEE) demonstrating a density (identified by arrow in (b)) on the mitral valve.

The patient is a 50-year-old man with a past medical history of arthritis who presented with mitral valve endocarditis ultimately requiring 6 months of antibiotics and a valvuloplasty. The patient initially presented to a community hospital for 3 days of painless right temporal field vision loss and was transferred to University Hospital in Newark. On initial evaluation, he denied nausea, vomiting, headache, or gait disturbances. He was working in maintenance at an apartment complex and had emigrated to the United States in 1991 from Guatemala. Initial workup including a computed tomography (CT) scan of the head and a magnetic resonance imaging (MRI) exam of the brain from a local community hospital showed multiple abscesses in the left frontal, occipital, and parietal lobes with leptomeningeal enhancement (Figures 1(a) and 1(b)). Despite his vision loss, the patient did not have other cranial nerve deficits, had full motor strength, and did not have pronator drift. He did not have a carotid bruit, Raccoon eye sign, or Battle's sign. He was evaluated by ophthalmology and found to have a right-sided visual field cut, but no significant macular changes. He was started on vancomycin, cefepime, and metronidazole for empiric coverage while undergoing further infectious workup and imaging. A CT chest/abdomen/pelvis demonstrated infarcts in the spleen and bilaterally in the kidneys (Figure 2). Transthoracic echocardiography (TTE) demonstrated a density on the mitral valve, and a follow-up transesophageal study (TEE) showed a 1.8 × 0.4 cm vegetation despite several sets of negative blood cultures (Figures 3(a) and 3(b)). He was treated in the hospital for 12 days before being discharged on empiric ceftriaxone and doxycycline for culture-negative endocarditis. Due to a lack of insurance coverage, the patient received ceftriaxone at an infusion center. For this reason, he received two grams of this antibiotic every 24 hours rather than the preferred twice-daily dosing for central nervous system infection.

Upon follow-up in the infectious diseases (ID) clinic three weeks later, the patient reported improved vision with a reduced visual field cut deficit on the right side. He was having back pain while sitting and standing up that improved with walking. He had negative serological testing for *Bartonella, Mycoplasma, Echinococcus, Coxiella, Aspergillus*, Lyme disease, ANA, and CCP, as well as a negative *Cryptococcus* antigen result. At the time, the differential still included a culture-negative endocarditis with *Staphylococcus* or *Streptococcus* or an endocarditis secondary to other agents such as *Brucella, Legionella, Abiotrophia, Cutibacterium,* or *Tropheryma whipplei*; as such, ceftriaxone and doxycycline were continued. At a follow-up visit 10 days later, the patient's symptoms were improving, and his labs were negative for *Brucella*. Antibiotics were continued while waiting for a repeat CT head, which showed reduced left frontal and parietal lobe lesions from the initial scan.

**Figure 4:**
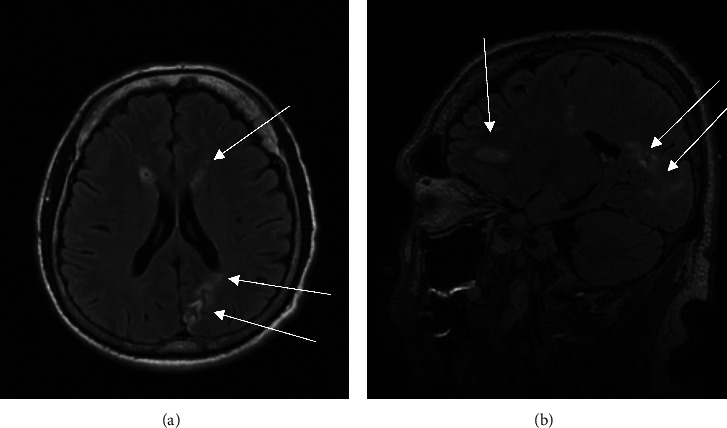
Repeat (a) axial and (b) sagittal views of MRI imaging after 12 weeks of antibiotic therapy demonstrated resolving left frontal, occipital, and parietal lobe abscesses.

One month after discharge from the hospital, the patient began to develop episodes of dizziness when rising from seated positions and sustained a fall. Given these symptoms in the setting of endocarditis, heart failure was suspected, though he had no lower extremity edema or rales on exam. TTE was repeated and now demonstrated an ejection fraction of 58% with a severely dilated left ventricle (LV), mildly increased LV wall thickness, and grade two diastolic dysfunction. He was seen by cardiology and cardiothoracic surgery. Carvedilol was started for heart failure, and evaluation was initiated for mitral valve replacement. He underwent repeat MRI brain after 12 weeks of antibiotic therapy, which demonstrated resolving left frontal lobe intraparenchymal abscess (Figures 4(a) and 4(b)), and later CT angiography of the head after 16 weeks of therapy, which demonstrated resolved abscesses. He ultimately underwent mitral valvuloplasty after 21 weeks of antibiotic therapy, with ceftriaxone, doxycycline, and rifampin continued until two weeks after surgery.

Pathology analysis after surgery showed a severely diseased mitral valve with a ruptured anterior leaflet requiring placement of a bioprosthetic valve. The patient's explanted valve was sent for 16S sequencing at the University of Washington. After surgery, he was without pain, dizziness, or new vision changes, and his sternotomy wound was healing well. Ultimately, 16S sequencing via PCR detected ribosomal DNA (rDNA) from *S. lugdunensis*. Results were indeterminate for *Bartonella*, *Legionella,* nontuberculous mycobacteria, *M. tuberculosis* complex, and *Tropheryma whipplei,* likely as a result of interfering bacterial templates from *S. lugdunensis*. After completing 6 months of antibiotics, the patient was able to resume work two months after surgery, and his only extant complaint was a few black spots in his visual field.

## 3. Discussion

Invasive *S. lugdunensis* infections are becoming increasingly common [1], and their incidence is likely to keep increasing with further adoption of molecular epidemiology detection tools like MALDI-TOF [2]. In addition to endocarditis, *S. lugdunensis* may present with skin and soft tissue infections, bone infections, and prosthetic joint infections [3]. *S. lugdunensis* is typically susceptible to most commonly used anti-Staphylococcal antibiotics [3, 4]. First-line treatment for most *S. lugdunensis* infections consists of oxacillin [3]; for endocarditis, the Infectious Diseases Society of America guidelines recommend treatment with a *β*-lactam agent and monitoring for any spreading of the infection [5]. Typically, *S. lugdunensis* endocarditis requires surgery with valve replacement in addition to antibiotics, with much higher need for surgery compared to *S. aureus* IE [3].

Early diagnosis of invasive *S. lugdunensis* infection is challenging because CoNS-positive cultures are often not sufficient to distinguish contamination or colonization from a clinically significant infection [3]. It remains unclear why blood cultures were negative in this case of an aggressive and disseminated endovascular infection; however, this could potentially be explained if the patient received active antibiotic therapy at the outside hospital prior to being transferred to our facility. While this exact scenario has not been well documented in previous literature, in one case study, a uterine cancer patient with *S. lugdunensis* urinary tract infection and associated neutropenic fever had negative blood cultures, although urine cultures grew the bacteria [6]. With more widespread use of molecular diagnostic tools like MALDI-TOF, cases of *S. lugdunensis* endocarditis that represent infection rather than contamination/colonization may be detected earlier, ideally avoiding delayed diagnosis and reducing patient morbidity.

## Data Availability

The clinical data used to support the findings of this case report have not been made available to protect patient privacy.
